# An Efficient Method of Modeling Material Properties Using a Thermal Diffusion Analogy: An Example Based on Craniofacial Bone

**DOI:** 10.1371/journal.pone.0017004

**Published:** 2011-02-11

**Authors:** Julian L. Davis, Elizabeth R. Dumont, David S. Strait, Ian R. Grosse

**Affiliations:** 1 Department of Engineering, University of Southern Indiana, Evansville, Indiana, United States of America; 2 Department of Biology, University of Massachusetts Amherst, Amherst, Massachusetts, United States of America; 3 Department of Anthropology, University at Albany, Albany, New York, United States of America; 4 Department of Mechanical and Industrial Engineering, University of Massachusetts Amherst, Amherst, Massachusetts, United States of America; Raymond M. Alf Museum of Paleontology, United States of America

## Abstract

The ability to incorporate detailed geometry into finite element models has allowed researchers to investigate the influence of morphology on performance aspects of skeletal components. This advance has also allowed researchers to explore the effect of different material models, ranging from simple (e.g., isotropic) to complex (e.g., orthotropic), on the response of bone. However, bone's complicated geometry makes it difficult to incorporate complex material models into finite element models of bone. This difficulty is due to variation in the spatial orientation of material properties throughout bone. Our analysis addresses this problem by taking full advantage of a finite element program's ability to solve thermal-structural problems. Using a linear relationship between temperature and modulus, we seeded specific nodes of the finite element model with temperatures. We then used thermal diffusion to propagate the modulus throughout the finite element model. Finally, we solved for the mechanical response of the finite element model to the applied loads and constraints. We found that using the thermal diffusion analogy to control the modulus of bone throughout its structure provides a simple and effective method of spatially varying modulus. Results compare favorably against both experimental data and results from an FE model that incorporated a complex (orthotropic) material model. This method presented will allow researchers the ability to easily incorporate more material property data into their finite element models in an effort to improve the model's accuracy.

## Introduction

Technological advances in medical imaging and software have facilitated the construction of geometrically complicated three dimensional representations of organic structures that are used in applications ranging from prototyping to computer-based analysis. In particular, these technological advances have allowed for more detailed studies of the skeletal system and its components using finite element (FE) models [1]–[28]. FE analysis (FEA) is a standard technique used by engineers to model the response of structures to applied boundary conditions (i.e. constraints and loads). For structural FEA, the material properties of the substances that comprise the structure are inputs for the model. By applying different material models (e.g., isotropic vs. orthotropic) to the same finite element model, investigators can study the effect of those material models on the response of geometrically complicated structures.

Previous methods for incorporating non-homogeneous moduli into FE models of biological structures have been developed [23], [29]. However, the collection of material property data and the precise application of those data to FE models of biological structures can be a time consuming and difficult process. To our knowledge, methods of easily including and controlling spatially variable material properties based on values measured from experiments in an FE model of a biological structure has not yet been presented.

Bone, for example, is often tested at discrete locations and has been modeled as a material ranging from isotropic to anisotropic depending on the length scale of observation [30]–[33]. As is always the case in modeling, it is desirable to employ simplifying assumptions regarding model inputs that allow efficient model construction while simultaneously ensuring adequate model accuracy. Therefore, detailed material data may not always be appropriate, or even available, for studies that use FE models. However, some research questions require high fidelity FE models that may be improved by incorporating detailed material data.

Strait *et al.* [23] investigated the effect of material models on the performance of an FE model of a macaque cranium (*Macaca fasicularis*). They quantified performance by comparing three strain measures collected *in vivo* (maximum shear strain, principal strain ratio, and maximum principal strain orientations) to those predicted by the FE model at eight locations on the skull. The analysis compared the performance of the FE model in four analyses in which assumptions about bone material properties ranged from coarse (a single set of isotropic material properties derived from human limb bones applied to the entire cranium) to precise (i.e., regionally varying orthotropic properties derived from an analysis of several macaque crania [34]). The results indicated that using an orthotropic material model whose properties were derived from and varied across the macaque cranium returned strain data that most closely approximated experimental data. While this study demonstrated the importance of using orthotropic material models to achieve results that most closely match experimental data, it came at the cost of painstaking and time consuming subdivision of the model into distinct material regions and creation of over thirty local coordinate systems representing the principal directions of the orthotropic material properties at various locations on the skull.

In an effort to minimize the time consuming process of applying orthotropic material models to FE models, Helgason *et al.* [29] presented a novel method of applying spatially-varying (i.e., non-homogeneous) elastic modulus throughout an FE model by exploiting the coupled thermal-structural analysis feature typically found in commercial FEA tools. Helgason et al. [29] created a voxel-based finite element model of a femur from computed tomography (CT) data. A Young's modulus value for each node of the voxel-derived FE mesh was computed using a nonlinear relationship between Young's modulus and bone ash density. Young's modulus values were obtained via a calibrated linear relationship with bone ash density from the CT scan. Finally, nodal temperature values were used as a surrogate for Young's modulus by assigning an arbitrary linear relationship between temperature and Young's modulus. The net result was temperature values for all nodes and a temperature-dependent Young's modulus material behavior model. The FE code then solved the structural problem. This method allows for precise control of material properties throughout an FE model and has the potential to significantly simplify the process of assigning complex material properties to bones. However, the approach requires a voxel-based finite element mesh and relies on the relatively low correlation of cortical bone density to Young's modulus; *r^2^* values range from a low of 0.24 [35] to a high of 0.69 [36].

In this paper we extend the method proposed by Helgason *et al.* [29] to any finite element mesh for which material property values are known only at specific locations. We take full advantage of the coupled thermal-structural functionality in commercial codes by modeling the distribution of Young's modulus as a thermal diffusion phenomenon. Specifically, we apply temperature values (which are linearly correlated to experimentally-measured values of Young's modulus) at seed nodes, define isotropic thermal material behavior, and solve the steady-state heat conduction problem. Once the finite element temperature solution is obtained, the coupled thermal-structural analysis as described above is executed. This method differs from Helgason *et al.* [29], who fully specified Young's modulus at every node according to CT scan data and therefore did not need to solve a thermal FE model to obtain the temperature distribution used to functionally grade Young's modulus. Our extension of Helgason *et al.*'s [29] technique provides a modulus distribution throughout the model based on heat flow paths through the material.

The goal of this study is to determine whether modeling bone using our thermal-structural approach can either improve or equal the predictions of maximum shear strain derived from a model in which different orthotropic material properties are defined for different regions: Regionally Orthotropic FE model. We do this by applying our thermal analogy method to the macaque finite element model of Strait *et al.* [23] under identical loading and boundary conditions. We implement 3 FE models that differ only in the material properties applied to each model. The Uniform Isotropic (UI) finite element model uses the same isotropic material properties through-out each anatomical region of the skull distinguishing only cortical from trabecular bone. The Regionally Isotropic (RI) finite element model also uses isotropic material properties in each anatomical region of the skull, however, the modulus and Poisson's ratio varied discretely between anatomical regions. Finally, the Thermally Graded (TG) finite element model allows modulus vary smoothly according to seed values and propagate though the FE model using heat paths. For this analysis we focus on maximum shear strain; complete experimental data are not available for principal strain ratios and principal strain orientation comparisons are highly qualitative [23]. Included with our results of maximum shear strain are the results obtained using the Regionally Orthotropic FE model by Strait *et al.* [23]. If our temperature dependent modulus method performs well, it will significantly reduce the time it takes to define and apply complex material properties to FE models.

## Results

**Figure 1:**
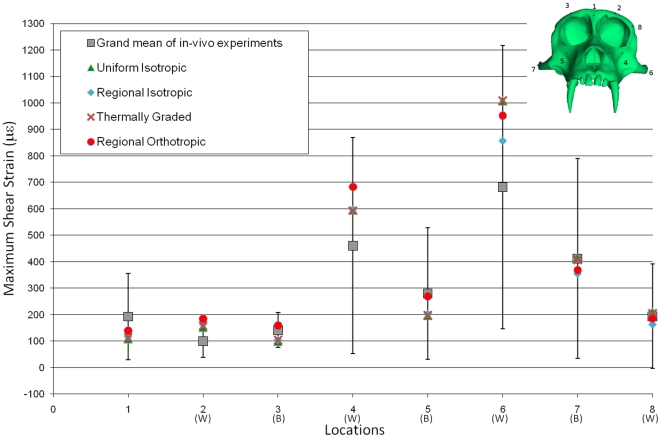
Experimental and FE model maximum shear strains. Maximum shear strains collected from each of the analyses are plotted against grand mean of experimental data. Also included in this figure are the shear strain results from the orthotropic material property FE model (Regional Orthotropic) used in the 2005 study of same model [23]. All other data are derived from this study. Working and balancing side data are labeled with (W) and (B), respectively. Descriptions for each location shown on the macaque skull (upper right) are as follows: 1) dorsal interorbital; 2) working side dorsal orbital; 3) balancing side dorsal orbital; 4) working side infraorbital; 5) balancing side infraorbital; 6) working side zygomatic arch; 7) balancing side zygomatic arch; 8) working side postorbital bar.

Shear strain from eight nodes that matched those used in Strait *et al.* in 2005 were identified on each of three finite element models: Uniform Isotropic, Regional Isotropic and Thermally Graded. Each material model is discussed further in the materials and methods section. We compared the strain results with experimental data collected from the literature and the FE results from the regionally orthotropic FE model used in Strait et al. in 2005. With one exception, predicted shear strain from all of the material models fell within the range of the experimental data. The exception was location number 2 (the superior rim of the orbit on the working side) in the regional orthotropic material model, where shear strain fell just outside of the range of the experimental data (Fig. 1). The results from the Uniform Isotropic and Regional Isotropic analyses are in basic agreement with those of Strait *et al.* [23], considering the slight over-estimation of shear modulus.

## Discussion

As noted by Strait *et al.* in 2005, the need for accurate and detailed material properties within a model is largely based on what research questions are being asked. In this and the 2005 study, the criterion for model performance was whether the model predicted strain that fell in the range of *in-vivo* data collected from several individuals, which spanned over 1000 microstrain. Given this wide range of variation, all material models we examined performed equally well. Note, however, that all of the models used material properties derived directly from macaque crania. The authors also examined the FE model using material properties derived from a different species and skeletal part (i.e. human limb bone), and in that case found a weaker correspondence between FE and *in vivo* data. Given that relatively few FE analyses of vertebrate skeletal structures employ material properties derived directly from the species and/or skeletal part of interest, the cautions outlined by Strait *et al.* [23] remain salient. In reference to the present study, a more fine scaled determination of whether one material model is superior to the others will require more tightly controlled experimental data. Ideally, one would compare the performance of different material models using a complete set of data on regional material properties, experimentally measured strains, and strains from an FE model derived from a single individual.

**Figure 2:**
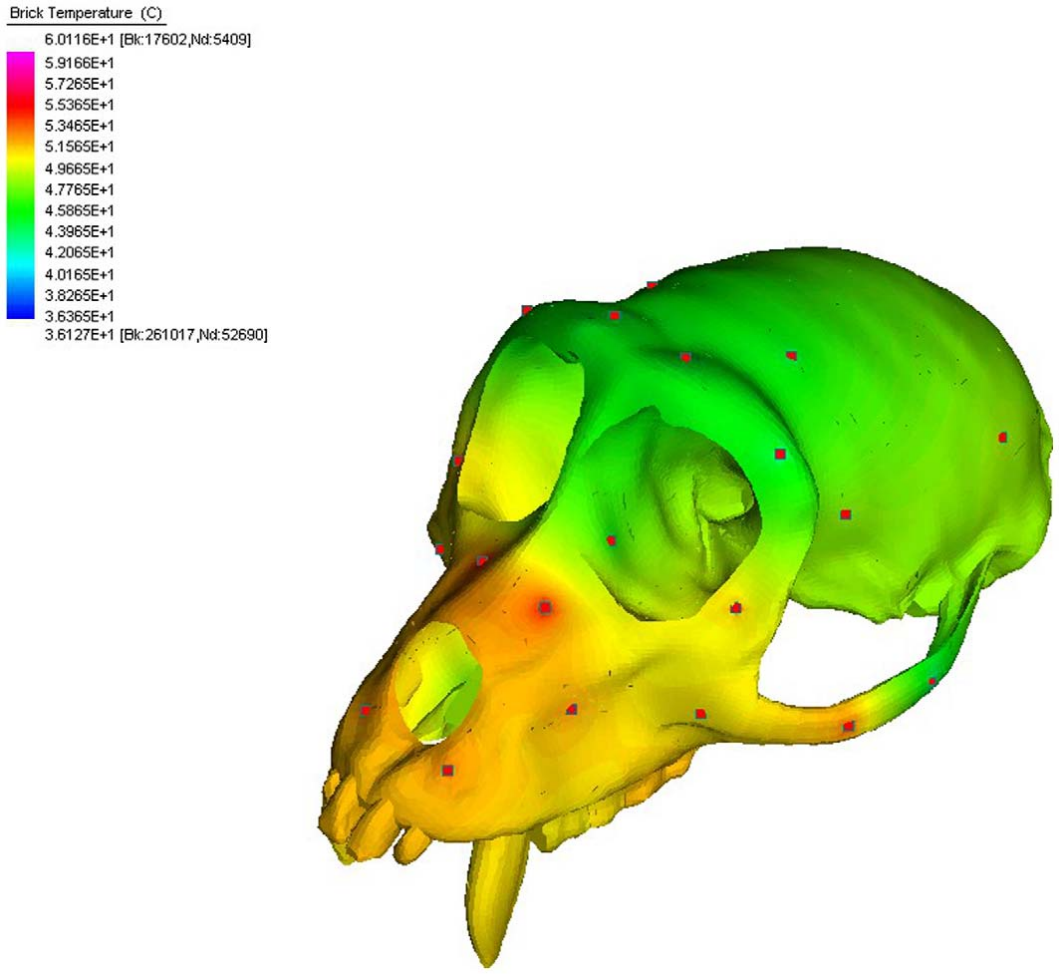
Steady state temperature distribution. Steady state temperature distribution throughout the skull is determined by heat conduction based on temperatures at seed points (red squares) on the skull. High temperatures in regions of the skull indicate high moduli according to the linear relationship shown in Figure 3.

In the absence of complete studies of single individuals, modeling bone as a functionally graded isotropic material is relatively easy and can be implemented in many commercial FE packages. In fact, most commercial FEA programs provide automated techniques for coupling the results of a thermal analysis to the subsequent structural analysis problem in which mechanical properties of the structure are temperature dependent. The method has the advantage that it does not require a voxel-based finite element model or rely on indirect measurements of Young's modulus. Instead, it applies a functionally graded material model based on known material values data collected from specific locations. Ideally, these data are gathered via direct physical tests, such as micro indentation or ultrasonic testing [37]–[38]. Another advantage of this method compared to an approach in which material properties are partitioned according to region (as in Strait *et al.* [22]–[25]) is that it avoids sudden, unrealistic transitions in those properties across the boundaries between parts (Fig. 2). These transitions create artifacts in the strain field that would not be observed using the thermal diffusion approach described here.

An assumption behind the thermally graded material model we propose is that the non-homogeneous distribution of material stiffness in a bone can be approximated by the solution of a heat conduction problem using the temperature field as a surrogate for Young's modulus. Although bone has been shown to have spatially variable modulus on macroscopic [32], [38] and microscopic scales [37], we are not claiming diffusion is the mechanism by which stiffness is propagated through bone, nor is it necessarily the appropriate descriptive model. We are simply using diffusion to spatially interpolate intermediate values between known values of measured modulus. We suggest that it is reasonable hypothesis to let the value of Young's modulus propagate throughout a specimen from values at specific points according to the physics of basic diffusion mechanics. Steady state heat conduction is governed by Laplace's equation, an equation encountered in mass transfer theory, fluid mechanics, elasticity, electrostatics and virtually all diffusion and potential field problems. Therefore it seems reasonable to apply here.

An inherent limitation to the proposed technique is the inability to functionally grade orthotropic material property values. While it might be possible to control spatially each of the nine independent orthotropic material constants using nine independent thermal analyses, the orientations of principal material axis must also be controlled spatially. Fundamentally, temperature is a scalar field (i.e. a zero^th^ order tensor), has no directional information and therefore fails to provide an analogy for functionally grading a 2^nd^ order tensor field (i.e. orthotropic material model) which requires specification of both values and directions throughout the domain. It should be noted that the Hounsfield's unit is also a scalar field in CT bone scans and therefore also lacks sufficient information for specifying non-homogeneous orthotropic material properties.

Temperature and static solution tools associated with the FE method can facilitate the incorporation of more experimental data into the FE models (i.e., local measurements of modulus from micro- or nano-indentation) and perhaps improve a model's performance. The thermal method we outline here provides a quick and reasonable means for modeling the spatial variation of isotropic material properties within geometrically complicated structures, such as bone. This type of material is called a functionally graded material. Based on available *in-vivo* data derived from several macaque crania (none of which represents the cranium that is the subject of the FE model), the method produces maximum shear strains that are not discernibly any less realistic than those produced by a regional orthotropic model, but at a significantly reduced cost in terms of time and effort. Because the thermally graded properties are isotropic whereas the true properties of craniofacial bone are orthotropic, the method developed here is not necessarily more precise than regional orthotropy (in particular, the shear modulus of craniofacial bone is being overestimated). However, the discrepancy in strain results produced by these models is sufficiently small that the benefit gained in terms of ease of modeling makes the thermally graded approach preferable with respect to many research questions.

## Materials and Methods

Details of FE model construction, loading and constraints are discussed in Strait *et al.* [23]. Briefly, the macaque skull FE model was constructed from over 300,000 polyhedron elements. The FE model was divided into 53 different sections to which 17 material properties were assigned independently. Masticatory muscle forces were estimated using muscle stress, physiological cross sectional areas and electromyography data collected from the literature. Muscle forces were applied to the skull and oriented to mimic static unilateral biting with the left upper first molar tooth. The FE model was constrained from rigid body motion at the temporomandibular joints and the left first molar.

We performed analyses using three material models ranging from simple to complex, applied to the same FE model: a Uniform Isotropic material model, a Regional Isotropic material model, and a Thermally Graded material model. In the UI model we modeled both cortical and trabecular bone as homogeneous isotropic materials (cortical: Young's Modulus (E)  = 17.3 GPa, Poisson's Ratio (ν)  = 0.28; trabecular: E = 0.64 GPa, ν = 0.28) [23]. In the RI model, we applied varying isotropic material properties to each of the 53 sections of the cranium corresponding to regions listed in Table 1. Homologous regions on the right and left sides of the cranium were assigned the same properties, and several neurocranial and basicranial regions were assigned the properties used in the UI analysis. Some of the regions have multiple parts, but all were assigned the same material property. For example, trabecular bone was modeled with the same uniform isotropic material everywhere it occurred.

**Table 1:** Regional isotropic material properties from Strait *et al.* [23].

Region	Young's Modulus	Poisson's Ratio
	GPa	
Trabecular Bone	0.64	0.28
Posterior Zygomatic Arch	12.5	0.28
Frontal Torus	13.1	0.25
Glabella	14.4	0.27
Medial Orbital Wall	14.6	0.36
Frontal Squama	14.9	0.31
Anterior Palate	15.3	0.34
P3–M1 Alveolus	16.7	0.25
Neuro- and Basi- crania	17.3	0.28
Root of Zygoma	17.9	0.34
Lateral Rostrum	18.1	0.25
Premaxilla	18.5	0.21
Posterior Palate	18.8	0.32
Postorbital bar	19.8	0.27
Dorsal Rostrum	19.9	0.22
M2–M3 Alveolus	20.6	0.27
Anterior Zygomatic Arch	20.8	0.26

Each FE model was divided into 17 different regions for which separate material properties could be assigned. Regions and corresponding material properties are listed here.

Note that the isotropic material properties employed here are not precisely the same as the properties employed by Strait *et al.* [23]. The authors erred in that the properties they employed were not truly isotropic. Rather, they used published values of Young's modulus, Poisson's ratio and shear modulus, but because bone is not an isotropic material, the values of these variables do not satisfy the conditions of isotropy. Nevertheless, the values used by the authors in 2005 are more precise than isotropic values, because isotropic values derived from Young's modulus and Poisson's ratio systematically overestimate the shear modulus of bone. Thus, the UI and RI analyses presented here are performed on models with a higher shear modulus than those in the corresponding analyses of Strait *et al.* published in 2005. Ultimately, however, the material properties used here did not result in maximum shear strains that differed meaningfully from those obtained by Strait *et al.* [23].

**Figure 3:**
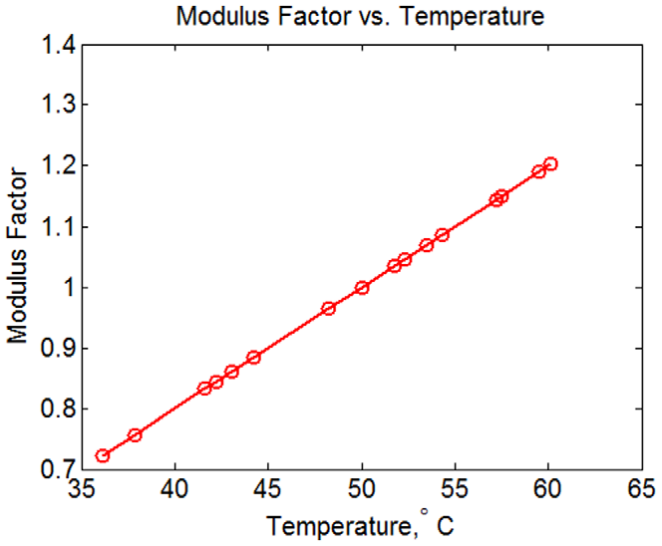
Modulus factor vs. temperature. This figure illustrates the linear relationship between modulus and temperature used in the Thermally Graded material model we developed in this study.

To implement the TG material model, we used the thermal analogy to functionally grade Young's modulus throughout the skull based on known values at specific points. This method was executed in several steps. The first step established a relationship between temperature and the moduli for all of the cortical bone used in the model (Table 1). We assigned the mid-range modulus of 17.3 GPa to a temperature of 50 degrees centigrade and determined the linear scaling factor to account for all the moduli used in the model (Fig. 2). We then assigned a temperature to a surface node at the approximate center of each of the 53 sections of the skull (Fig. 2) according to the moduli listed in Table 1 and the modulus versus temperature relationship shown in Figure 3.

In order to obtain the steady-state temperature distribution throughout the structure due to temperature values applied at specific points, thermal conductivities (k) were assigned to the materials used to model the structure. Thermal conductivity, measured in Watts per meter-Kelvin, is a measure of a material's ability to conduct heat through the material. Metals for example, have a relatively high thermal conductivity, between 10 and 400 W/(m-K) [39]. Bone tends to have lower thermal conductivity, between 0.2 and 0.5 W/(m-K) [40]–[42]. In this study the thermal material properties are simply a means by which modulus is propagated through the model via a finite element solution to the heat conduction equation. The steady-state temperature distribution within the structure, assuming constant thermal boundary conditions, is not affected by changes in the thermal conductivity value. Therefore, each material in the FE model was arbitrarily assigned a thermal conductivity of 1.0 W/(m-K). If one is interested in thermally induced strains as well as mechanical strains, a coefficient of thermal expansion (α), measured in strain per Kelvin, must be non-zero. However, we were only interested in mechanical strain, so assigned the coefficient of thermal expansion a value of zero.

We solved the steady state temperature problem using Strand7 finite element software (Strand7 Version 2.4, Sydney, Australia), by allowing temperature to propagate through the skull based on the temperatures we assigned to seed nodes (Fig. 2). Note that because temperature is the primary nodal variable solved for in a finite element thermal analysis, the resulting temperature field has 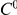
continuity across all inter-element boundaries and will vary within each element based on each element's interpolating polynomials (i.e., shape functions). A function with 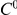 continuity is continuous. A function that has 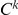
continuity, where *k* is a positive integer, is continuous, as are all its derivatives up to and including its *k^th^* derivative. For example, for a four-noded tetrahedral element the temperature field within an element is interpolated using linear polynomials in terms of coordinates x, y, and z from nodal values.

In the next step of the analysis, we imported the temperature solution back onto the solid model, thereby assigning temperatures (and modulus) to all of the nodes within the model. We then solved the structural temperature-dependent-modulus problem. The solution process employed by the solver required the computation of each element's stiffness matrix. The stiffness matrix for an element is related to both the shape and size of the element, as well as to its material stiffness. The element's non-uniform temperature field enables the elastic modulus within each element to vary continuously according to its prescribed temperature dependency. Finite element programs that support temperature-dependent elastic modulus and a corresponding initial temperature field take this into account when computing each element's stiffness matrix.
